# Genetics, Mechanisms, and Therapeutic Progress in Polyglutamine Spinocerebellar Ataxias

**DOI:** 10.1007/s13311-018-00696-y

**Published:** 2019-01-03

**Authors:** Ronald A.M. Buijsen, Lodewijk J.A. Toonen, Sarah L. Gardiner, Willeke M.C. van Roon-Mom

**Affiliations:** 1 Department of Human Genetics, LUMC, P.O. Box 9600, 2300 RC Leiden, The Netherlands; 2 Department of Neurology, LUMC, P.O. Box 9600, 2300 RC Leiden, The Netherlands

**Keywords:** Spinocerebellar ataxia, SCA, polyglutamine disorders, gene therapy, stem cell-based therapy, antisense oligonucleotides

## Abstract

Autosomal dominant cerebellar ataxias (ADCAs) are a group of neurodegenerative disorders characterized by degeneration of the cerebellum and its connections. All ADCAs have progressive ataxia as their main clinical feature, frequently accompanied by dysarthria and oculomotor deficits. The most common spinocerebellar ataxias (SCAs) are 6 polyglutamine (polyQ) SCAs. These diseases are all caused by a CAG repeat expansion in the coding region of a gene. Currently, no curative treatment is available for any of the polyQ SCAs, but increasing knowledge on the genetics and the pathological mechanisms of these polyQ SCAs has provided promising therapeutic targets to potentially slow disease progression. Potential treatments can be divided into pharmacological and gene therapies that target the toxic downstream effects, gene therapies that target the polyQ SCA genes, and stem cell replacement therapies. Here, we will provide a review on the genetics, mechanisms, and therapeutic progress in polyglutamine spinocerebellar ataxias.

Autosomal dominant cerebellar ataxias (ADCAs) are a group of neurodegenerative disorders characterized by degeneration of the cerebellum and its connections [1, 2]. All ADCAs have progressive ataxia as their main clinical feature, frequently accompanied by dysarthria and oculomotor deficits. The ADCAs of which the causative gene has been identified belong to the spinocerebellar ataxias (SCAs). The SCAs are numbered in chronological order of discovered gene locus with SCA47 being the most recent SCA reported [3–5]. However, around 1 third of families with ADCA remain without a genetic diagnosis [6, 7]. Based on a meta-analysis of prevalence studies, the prevalence of SCAs is estimated to be approximately 1–5:100,000 [7, 8], but can vary widely between different geographical and ethnical groups (see Fig. 1) [17]. The highest prevalence rates in population-based studies were found in Portugal (5.6:100,000 [6]), in Norway (4.2:100,000 [19]), and in Japan (5.0:100,000 [20]).

**Fig. 1:**
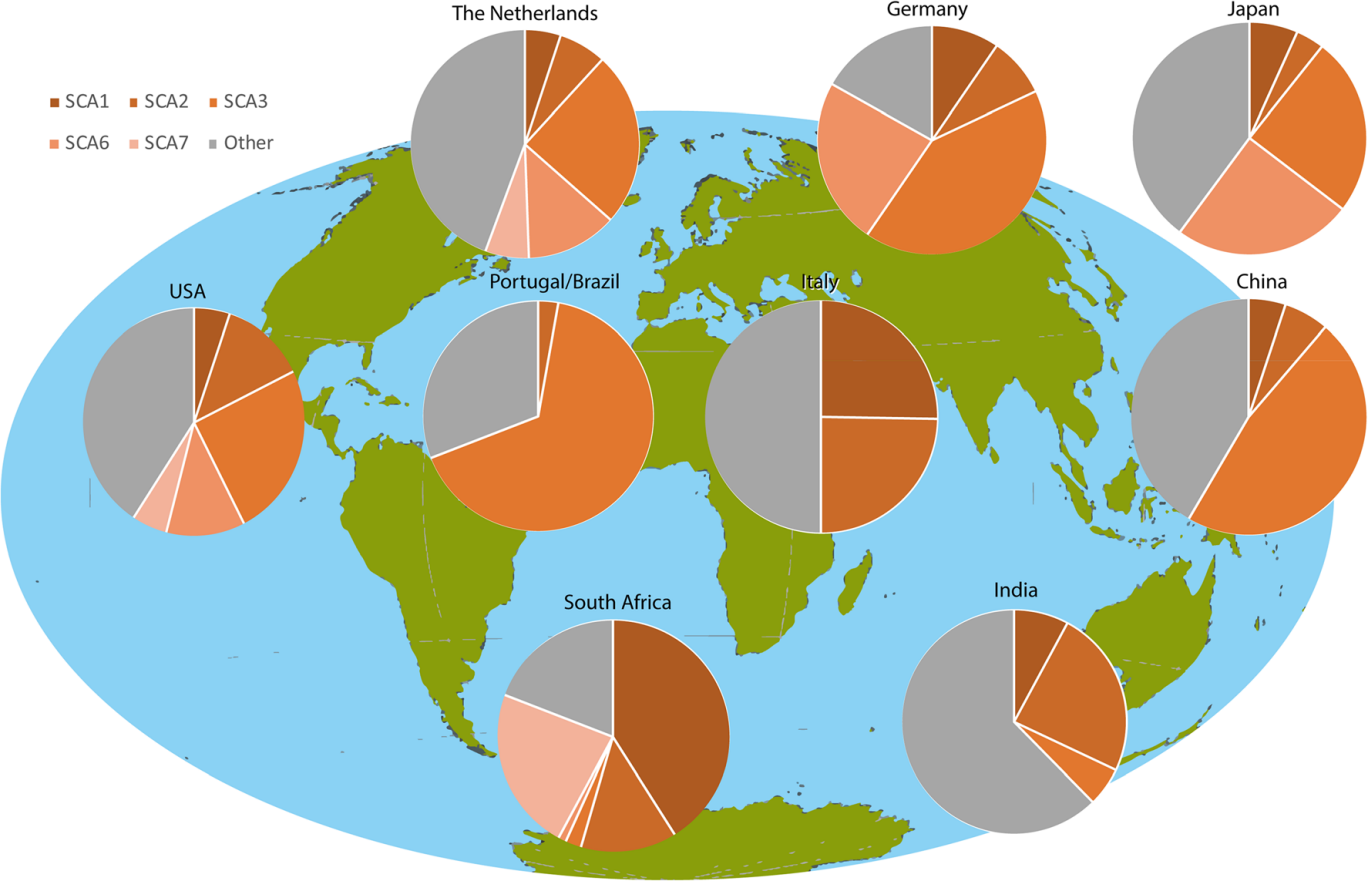
Worldwide prevalence of polyglutamine spinocerebellar ataxias. In the ADCA families investigated, no SCA17 was identified. The Netherlands [8]; Germany [9]; Japan [10]; the USA [11]; Portugal/Brazil [12]; Italy [13]; China [14]; South Africa [15]; India [16]. Figure based on Schols et al. [17] and adapted from Bird [18] (GeneReviews)

The most common SCAs are 6 polyglutamine (polyQ) SCAs. These diseases are all caused by a CAG repeat expansion in the coding region of a gene. For each SCA, the causative gene is different (see Table 1) [21–41]. The expanded CAG repeat in the DNA is translated into an expanded repeat of glutamine amino acids (Qs) in the respective ataxin proteins, hence the term polyQ SCA. In general, an inverse correlation exists between the length of the expanded polyQ stretch and the age of symptom onset [42, 43]. Although the exact pathogenic mechanism for each polyQ SCA is not fully understood, the known mechanisms have been extensively reviewed previously (see review [44]). In short, data suggests that the disruption of the native protein functions and the toxic gain of function conveyed by the expanded polyQ stretch [45] are interrelated and disrupt several common cellular processes contributing to disease pathology. These disruptions include transcriptional deregulation, RNA toxicity, toxicity caused by repeat-associated non-ATG (RAN) translation peptides, dysregulation of the ubiquitin proteasome system, and autophagy [46]. In addition, the altered protein confirmation caused by the expanded polyQ sequence leads to the formation of large insoluble protein aggregates containing the expanded diseased protein mainly in neurons of the cerebellum. Whether these large aggregates, a hallmark of all polyQ SCAs, are neurotoxic or neuroprotective is still under debate [47]. However, for most polyQ SCAs, soluble mono- or oligomer fragments are thought to be the main toxic entity [48, 49]. Together, these disrupted cellular processes cause perturbations of the intracellular homeostasis and eventually lead to the death of neuronal cells, inducing the characteristic neurodegenerative symptoms of the polyQ SCAs. Despite their similarities, the different polyQ SCAs have distinct clinical and neuropathological features described in the sections below.

**Table 1:** Characteristics of polyglutamine disease-associated genes

Disease	PDAG	Locus	Protein	Repeat	CAG repeat number		
					Normal	Intermediate	Pathological
SCA1	*ATXN1*	6p22.3	Ataxin-1	(CAG)*n*(CAT)*n*(CAG)*n*^a^	6–35 (6–44^a^)	36–38	39–91 (45–91^a^)
SCA2	*ATXN2*	12q24.12	Ataxin-2	[(CAG)*n*CAA(CAG)*n*]*n*^b^	14–31	32	33–500
SCA3	*ATXN3*	14q32.12	Ataxin-3	(CAG)2CAA AAG CAG CAA(CAG)*n*	11–44	45–59	60–87
SCA6	*CACNA1A*	19p13.13	CACNA1A	(CAG)*n*	4–18	19	20–33
SCA7	*ATXN7*	3p14.1	Ataxin-7	(CAG)*n*	4–19	28–33	34–460
SCA17	*TBP*	6q27	TBP	[(CAG)*n*(CAA)*n*(CAG)*n*]	25–40	–	41–66

CAG = cytosine-adenine-guanine; PDAG = polyglutamine disease-associated genes; CACNA1A = calcium channel, voltage-dependent P/Q type, α1A subunit; TBP = thymine-adenine-thymine-adenine (TATA) box-binding protein; SCA = spinocerebellar ataxia

^a^Range if CAT trinucleotide repeat interruptions are present

^b^Could be interrupted by 1-4 CAA trinucleotide repeats

## SCA1

The prevalence of SCA1 worldwide is around 1 to 2 per 100,000 individuals [50]. A large variance exists in the number of individuals with ADCAs that are confirmed to be SCA1. The prevalence ranges from no cases reported in a study among Korean patients [51] to essentially 100% of all patients in an Eastern Siberian study, where SCA1 was the only type of ADCA identified in the ethnic Sakha population [52].

The main symptom in SCA1 is progressive cerebellar ataxia characterized by disturbances in balance and gait. Oculomotor movements are also affected [53, 54]. Furthermore, patients frequently suffer from pyramidal, extrapyramidal, and bulbar symptoms [1]. As the disease advances, muscle atrophy arises. Cognitive symptoms have been reported in the final stages of the disease with impaired executive function being the most common defect [55]. Disease onset is usually in the third or fourth decade of life and shows a more rapid disease progression than the other polyQ SCAs. Affected individuals eventually develop respiratory failure, which is the main cause of death [56]. Interval from onset to death varies from 10 to 30 years. Individuals with juvenile onset show a more rapid disease progression and more severe disease [17]. The neuropathology in SCA1 shows severe atrophy of the cerebellum and brainstem. In addition, degeneration of the frontal, temporal, and parietal cerebral lobes is seen with the basal ganglia, midbrain, and thalamus also affected [57].

SCA1 is caused by an expanded CAG repeat in the *ATXN1* gene [21]. Normal alleles have 6 to 39 repeats, while pathological repeats have 39 to 91 repeats [21, 22]. Ataxin-1 is a protein of 98 kDa, and research has shown that this protein is required for cognitive function, motor coordination, as well as the processing of β-amyloid precursor protein (APP) [58–60]. Furthermore, ataxin-1 can act as a transcriptional repressor [61–63]. The ataxin-1 protein is involved in transcriptional regulation and RNA metabolism [64] and has a role in extracellular matrix (ECM) remodeling through formation of a transcriptional repressor complex with Capicua [65].

## SCA2

Around 0.1 to 5.8 per 100,000 people suffer from SCA2 [66] with 6% to 33% of ADCA patients being diagnosed with SCA2. Lowest percentages were reported in Japan (5.9%) [67], while the highest percentages were reported in Korea (33%) [68].

SCA2 is characterized by progressive cerebellar ataxia, dysarthria, and oculomotor deficits, including abnormally slow saccades and nystagmus [69]. Patients can also suffer from intention and postural tremors, myoclonus, Parkinsonism, sleep disturbances, autonomic dysfunction, and initial hyperreflexia followed by peripheral neuropathy with hyporeflexia or areflexia [70]. In addition, different studies have reported late cognitive deficits [71] as well as psychiatric symptoms [72]. The onset of symptoms is usually in the fourth decade of life with a disease duration of 10 to 15 years [73]. Onset before the age of 20 years correlates with a more aggressive disease course [74]. SCA2 presents with atrophy of the cerebellum characterized by severe loss of cerebellar Purkinje cells (PCs) while the deep cerebellar nuclei (DCN) are relatively spared. Other major affected brain regions are the pons, medulla oblongata, and spinal cord. Pronounced neuronal loss is also seen in the cerebral cortex, basal forebrain, basal ganglia, and midbrain [57].

SCA2 is caused by an unstable expansion of a CAG/CAA repeat in the *ATXN2* gene [75, 76]. Normal alleles have 14 to 31 repeats, while pathological repeats have 33 to 500 repeats [23–25, 75, 76]. The protein associated with SCA2 is the ataxin-2 protein. Ataxin-2 is a protein of 145 kDa with its main function in RNA metabolism, regulation of translation, stress granule formation, and P-body formation [77]. Of particular interest are the interactions of ATXN2 with polyadenylate-binding protein (PABP) and transactive response (TAR) DNA-binding protein 43 (TDP43), each of which also binds directly to RNA. One hypothesis is that the polyQ tract length in ataxin-2 impairs interactions with PABP [78] and TDP43, thereby contributing to SCA2 pathogenesis, as well as the risk of amyotrophic lateral sclerosis [79].

## SCA3/Machado–Joseph Disease

SCA3, also known as Machado–Joseph disease (MJD), is, in many populations, the most common polyQ SCA with an estimated prevalence of 1 to 2 per 100,000 individuals with significant geographical and ethnic variations. Prevalence differs greatly per population investigated with percentages of SCA3 diagnosed patients ranging from 0 to 1% to 58% among ADCA patients. The lowest percentages were reported in Italy (approximately 1%) [13] and Finland (~0%) [80], while the highest percentages were seen in Taiwan (47.3%) [81] and Portugal (57.8%) [82].

General SCA3 symptoms include oculomotor symptoms, cranial nerve deficits, sleep disturbances, involuntary weight loss, and autonomic problems [83]. Furthermore, SCA3 patients are known to suffer from mild cognitive impairments [84]. Disease duration is reported to range between 6 and 29 years [85]. Clinically, SCA3 can be further divided into 4 subtypes [83, 86]. SCA3 type 1 has an early age of onset between 10 and 30 years and is characterized by pyramidal and extrapyramidal symptoms with cerebellar ataxia being less prominent. Type 2 is the most common SCA3 subtype with an age of onset between 20 and 50 years. This type involves distinct cerebellar ataxia, dysarthria, and pyramidal symptoms. Age of onset of type 3 is typically later (40–75 years). Symptoms include cerebellar ataxia, but additionally, peripheral neuropathy leading to muscle atrophy and areflexia can be present. Type 4 has a variable age of onset and is characterized by Parkinsonism [87].

SCA3 affects various different regions of the brain and spinal cord, although the cerebellar cortex and olivary nuclei are relatively spared. The brain weight of SCA3 patients is significantly reduced, and neuronal cell loss is most prominent in the cerebellar dentate nucleus and substantia nigra. Neurodegeneration can also be seen in the cerebral cortex, pons, medulla oblongata, thalamus, and basal ganglia. In the spinal cord, the dorsal root ganglia, dorsal nuclei, and anterior horn are affected [57, 88].

SCA3 is caused by a CAG trinucleotide repeat expansion in the *ATXN3* gene [26]. Normal alleles range from 12 to 44 CAG repeats, while pathological alleles have 60 to 87 CAG repeats [26, 27]. The approximately 42 kDa SCA3-associated protein, the ataxin-3 protein (ATXN3), is ubiquitously expressed. This protein contains an N-terminal Josephin domain that displays ubiquitin protease activity and a C-terminal tail with several ubiquitin interacting motifs. ATXN3 functions mainly as a de-ubiquitinating enzyme [89].

## SCA6

The overall prevalence of SCA6 is between 0.02 and 0.031 per 100,000 individuals [90]. Around 12% of the families with ADCAs are diagnosed with SCA6 with the lowest percentage reported in Germany (1%) [91] and the highest in Japan (31%) [92].

SCA6 is considered a *pure* cerebellar ataxia. Primary symptoms include slow progressive cerebellar ataxia, dysarthria, and nystagmus. In 40 to 50% of the patients, pyramidal symptoms such as hyperreflexia and extensor plantar responses have been noted. Furthermore, signs of basal ganglia involvement including dystonia and blepharospasm are reported in 25% of the cases. Cognitive function is usually intact. The mean age of onset of SCA6 is between 43 and 52 years (range 19–71 years). Although the disease causes a high morbidity, the lifespan of patients is usually unaltered [93, 94].

In SCA6, widespread neurodegeneration comparable to that of SCA1, SCA2, SCA3, and SCA7 is present, but less severe. Degeneration mainly occurs in the cerebellum. Particularly, the cerebellar Purkinje cells are affected [95]. Microscopic pathology is more widespread in the thalamus, midbrain, pons, and medulla oblongata [57].

SCA6 is caused by a CAG expansion in the *CACNA1A* gene [28]. Normal alleles range from 4 to 18 CAG repeats, while expanded repeats range from 20 to 33 CAGs [28–31]. *CACNA1A* encodes 2 structurally unrelated proteins with distinct functions: the α1A subunit of P/Q-type voltage-gated calcium channel and α1ACT, a newly recognized transcription factor, with the polyglutamine repeat at the C-terminal end [96]. The α1A subunit of P/Q-type voltage-gated calcium channel is highly expressed in the Purkinje cells [97].

## SCA7

The prevalence of SCA7 is less than 1 per 100,000 individuals [11, 98]. Adult-onset SCA7 is characterized by progressive cerebellar ataxia, slowed ocular saccades, dysarthria, dysphagia, and pyramidal symptoms, such as hyperreflexia and spasticity [99]. Retinal degeneration is a distinctive feature of SCA7 with progressive cone-rod dystrophy leading to eventual blindness [100]. A decline in cognitive function and episodes of psychosis have also been reported [101]. The mean age of onset is about 32 years (range of 1–72 years) [102]. Clinical features differ between patients with an adult onset and patients with an onset in infancy or early childhood with the disease starting in childhood showing a more rapid and aggressive progression within a few years. When disease symptoms first appear later in life, the disease progresses more slowly and the degree of disability will vary accordingly [103].

In SCA7, extensive degeneration is observed in the retina. Furthermore, severe atrophy of the cerebellum and the brainstem is observed as well as neurodegeneration in the cerebral cortex, basal ganglia, thalamus, and midbrain [57].

SCA7 is caused by a CAG repeat expansion in the *ATXN7* gene [32]. Normal SCA7 alleles range from 4 to 17 CAG repeats. Alleles bearing 28 to 33 repeats can give rise to disease alleles but are not associated with a SCA7 phenotype. Disease alleles with 34 to 460 CAG repeats have been reported [32–35]. The expanded polyQ protein in SCA7, the ataxin-7 protein (ATXN7), was found to associate with microtubules and to be involved in the cytoskeletal regulation [104]. Furthermore, this protein is an integral component of the mammalian SPT3-TAF9-ADA-GCN5 acetyltransferase (STAGA) transcription coactivator complex and mediates the direct interaction of STAGA with the CRX transactivator of photoreceptor genes [105].

## SCA17

Worldwide, less than 100 families with SCA17 have been reported and the prevalence is less than 1 in 100,000 individuals [106, 107].

SCA17 is also known as Huntington disease-like 4, and symptoms of this disease indeed resemble Huntington disease (HD). SCA17 presents itself with cerebellar ataxia, pyramidal signs, and involuntary movements including chorea and dystonia. Parkinsonism has also been reported. Furthermore, SCA17 patients are known to suffer from psychiatric symptoms, as well as dementia [108, 109]. The mean age of onset is 35 years with a broad range of 3 to 75 years, and the mean disease duration of about 20 years [102, 110].

SCA17 shows a mild global atrophy of the brain. Neurodegeneration is seen in the cingulate and para-hippocampal gyri, striatum, ventral thalamic nuclei, cerebellar Purkinje cell layer and dentate nucleus, substantia nigra, and inferior olive [57].

SCA17 is caused by an expanded CAG repeat in the TATA-binding protein (TBP) gene [111]. The repeat in *TBP* can generally be divided into 5 regions including 2 polymorphic (CAG)*n* stretches: (CAG)3 (CAA)3′(CAG)*n*′CAA CAG CAA′ (CAG)*n*′CAA CAG where the CAA triplets also code for a glutamine amino acids [112]. Normal SCA17 alleles contain 25 to 40 repeats while alleles with 41 to 49 repeats have incomplete penetrance. Alleles with 50 or more repeats were reported to have a full penetrance [36–38]. The SCA17-associated protein TBP is an important component of the initiation complex of eukaryotic RNA polymerases [113]. TBP is 1 of several transcription factors that form a preinitiation complex with RNA polymerase II, and this step is essential for initiating transcription [112].

## Therapeutic Progress

Currently, no curative treatment is available for any of the (polyQ) SCAs. However, several compounds and treatment strategies that aid in improving the quality of life exist. Readers are directed to more comprehensive reviews on the symptomatic treatment elsewhere [43, 114–117]. The aim of this review is to provide an overview of potential treatments for the polyQ SCAs that can be divided into pharmacological and gene therapies that target the toxic downstream effects, gene therapies that target the polyQ SCA genes, and stem cell replacement therapies.

## Pharmacological Therapies

Increasing knowledge on the pathological pathways of the polyQ SCAs has provided promising therapeutic targets to potentially slow disease progression. Since the polyQ SCAs are rare, targeting a disease mechanism that is present in several polyQ SCAs would provide a therapeutic approach applicable to more than 1 disease. However, therapeutic targets that are more specific to particular polyQ SCAs are also discussed (Table 2).

**Table 2:** Pharmacological compounds

Compound	Mechanism	Model system	Outcome	Reference
Dantrolene	Stabilization of intracellular calcium signaling	MJD84.2 SCA3 mouse	Prevented neuronal cell loss and improved motor phenotype	[118]
Temsirolimus	Autophagy induction	SCA3 mouse line 70.61	Reduces the number of aggregates in the mouse brain, decreases levels of cytosolic soluble mutant ataxin-3, and improves motor performance	[119]
Sodium butyrate	HDAC inhibitor, reversal of transcriptional downregulation	Ataxin-3-Q79 transgenic mice	Reversed histone hypoacetylation/transcriptional, ameliorated neurological phenotypes, and improved survival	[120]
H1152	Rock inhibitor, ataxin-3 downregulation	Ataxin-3-Q79 transgenic mice	Reduction mutant ataxin-3 protein level in the brain, improved motor phenotype	[121]
Caffeine	Nonselective adenosine receptor antagonist	Lentiviral-induced SCA3 mice	Reduction in ataxin-3 inclusions, cell injury, and striatal degeneration	[122]
17-DMAG	Hsp90 inhibitor but may act through autophagy induction	CMV MJD135 SCA3 mice	Delay in motor deficit progression and rescue coordination deficit	[123]
Lithium chloride	Autophagy induction	CMVMJD135 SCA3 mice	No overall beneficial effects	[124]
Citalopram	Serotonin reuptake inhibitor	CMVMJD135 SCA3 mice	Reduced ataxin-3 neuronal inclusions and ameliorated motor symptoms	[125]
Valproic acid	HDAC inhibitor	CMVMJD135 SCA3 mice	Limited effects on motor deficits; no effects on ataxin-3 inclusions	[126]
Combination of temsirolimus and lithium chloride	Autophagy induction	CMVMJD135 SCA3 mice	Deleterious effect; no improvement in neurological symptoms; induced neurotoxicity induced	[127]
Riluzole	Inhibition of glutamate release	Inducible SCA3 mouse	No improvement in motor deficits	[128]
Caloric restriction or resveratrol	SIRT1 activation, autophagy activation	Lentiviral-induced SCA3 mice	Amelioration of motor deficits and neuropathology	[129]
Lithium chloride	Induction autophagy + inhibition of GSK3β activity	SCA3 *Drosophila*	Prevented eye depigmentation, alleviated locomotor disability, and extended lifespan	[130]
Calpeptin	Inhibition of calpain cleavage	SCA3 iPSC model	Prevented ataxin-3 aggregate formation in neurons	[49]
Interferon-β	Induces expression PML protein, degrades mutant ataxin-7	SCA7(266Q/5Q) knock-in mice	Reduction of mutant ataxin-7 in neuronal inclusions; improvement in motor coordination	[131]
Granulocyte–colony-stimulating factor	Upregulating chaperones and autophagy	SCA17 mice	Improved motor coordination; reduced cell loss	[132]
Lithium carbonate	Speculated to correct gene expression changes	SCA1 mice (154Q)	Slowed neurodegeneration and improved motor coordination but did not improve lifespan	[133]

### Protein Clearance Potentiation

Cellular homeostasis is maintained through a delicate balance between protein synthesis and protein degradation. Neurons are particularly dependent on efficient protein degradation mechanisms, 1 of which is autophagy [134]. Indeed, autophagy is essential for maintaining neuronal health, and autophagic dysfunction is involved in many neurodegenerative disorders [135] since loss of autophagy can result in disruption of neuronal function and neurodegeneration [136]. For the polyQ SCAs, the misfolded mutant polyQ proteins present a major challenge to cellular protein quality control systems. For this reason, enhancing autophagy is considered a viable therapeutic strategy, and a variety of compounds have been tested to this end for the SCAs as well as several other neurodegenerative diseases [137, 138].

### Pharmacological Stimulation of Autophagy

One compound that has been extensively researched is lithium. Lithium has pleiotropic effects and can act on multiple pathways, but autophagic potentiation is 1 of the effects researched in the context of neurodegeneration. For instance, lithium chloride has been investigated in Alzheimer disease, amyotrophic lateral sclerosis, and Parkinson disease (PD) [139]. For SCA3, lithium chloride was tested in a *Drosophila* model, where the eye depigmentation and locomotor disability were successfully reduced. The observed effect was in part attributed to GSK3β/shaggy inhibition, which, in turn, perturbs the Wnt pathway [130]. However, the authors concluded that lithium clearly had additional beneficial effects that could not be solely explained by changes in this pathway [130]. In SCA1 knock-in (KI) mice, lithium treatment reduced neurodegeneration, improved motor symptoms, and restored cerebellar ascorbate levels [133, 140]. However, other studies have questioned the suitability of lithium for treatment of the polyQ SCAs. For instance, in SCA1 mice, life expectancy of mice was not improved despite improved motor function [133], suggesting that the underlying pathology was not sufficiently addressed through lithium treatment. Other research in SCA3 mice was unable to show any beneficial effect of lithium on a range of behavioral tests altogether, despite activation of autophagy [124]. Finally, a phase 2, double-blind, placebo-controlled trial testing lithium carbonate in 62 SCA3 patients was performed. The investigators concluded that lithium was well tolerated, but no effect on the Neurological Examination Score for Spinocerebellar Ataxia (NESSCA) was detected [141]. A clinical trial with lithium was also performed in 20 SCA2 patients, where only a significant effect on the Beck Depression Inventory could be established, but not on the Scale for the Assessment and Rating of Ataxia (SARA) score [142]. Based on these clinical trials, lithium treatment of the polyQ SCAs is currently not very well supported. More and larger clinical trials would be required to give a definitive decision on the suitability of lithium for SCA treatment [143], though major beneficial effects of lithium treatment for SCAs seem unlikely based on current knowledge.

Though lithium has thus far not been very successful as potential SCA treatment, potentiation of autophagy may still be a useful therapeutic strategy. Other compounds and means to stimulate autophagy have also been tested in preclinical SCA research. For instance, temsirolimus, through inhibition of mammalian target of rapamycin (mTOR), can potentiate autophagy and reduce toxicity in HD mouse models [144]. Temsirolimus was administered to SCA3 mice, where an improvement in motor phenotype was established and the number of aggregates in the mouse brain was also reduced [119]. However, when tested in combination with lithium chloride, no improvement in any parameter could be seen in SCA3 mice. In fact, only deleterious neurotoxic effects of the treatment were seen [127]. Whether the negative treatment effect was due to a toxic interactive effect of the 2 drugs or, potentially, an excessive degree of autophagic potentiation remains unclear [127]. To our knowledge, no clinical trials with temsirolimus have been performed for any of the SCAs.

### Other Strategies to Induce Autophagy

Apart from the more traditional small molecule-based therapeutics, autophagy can be stimulated through other, more indirect methods. For instance, caloric restriction is a potent inducer of autophagy and is hypothesized to be a potential strategy to delay neurodegenerative disease progression [145]. Caloric restriction indeed resulted in marked improvement in motor phenotype and neuropathology when performed in SCA3 mice [129]. The authors also show that the disease alleviation is mediated by the SIRT1 protein, a deacetylase found to slow aging [129]. Trehalose, an alpha-linked disaccharide, has been shown to clear accumulated proteins by activating autophagy, primarily in an mTOR-independent manner [146]. In SCA17 cell models and a SCA17 mouse model, nuclear protein aggregation was significantly reduced after trehalose treatment. Furthermore, gait behavior and motor coordination were significantly improved in the trehalose-treated SCA17 mice [147]. Moreover, in SCA3 cell models, protein aggregation was significantly prohibited by trehalose analogues [148]. A phase 2, open-label clinical trial has being conducted in SCA3 patients, but no results have been published yet.

Lastly, Beclin-1, an important initiator protein of autophagy [149], was found to be reduced in a SCA3 rat model and brain material from patients [150]. Using lentiviral expression, the researchers overexpressed Beclin-1, resulting in autophagic flux. As a result, mutant ataxin-3 was cleared more efficiently and neuroprotective effects were established [150]. Research using the Hsp90 inhibitor 17-DMAG in the CMV MJD mouse model for SCA3 resulted in rescue of the neuropathology and accompanying alleviation of the motor deficits. Somewhat unexpectedly, researchers did not find evidence for induction of heat shock proteins but instead found induction of Beclin-1. It was hence speculated that the beneficial effect of 17-DMAG may occur through autophagic potentiation [123]. Similarly, granulocyte–colony-stimulating factor was assessed as a potential therapy in SCA17 mice. The treatment reduced insoluble mutant TBP protein, and reduced Purkinje cell loss, resulting in improvement of the motor phenotype. As Beclin-1 and other autophagic factors were increased, this suggests that the beneficial effect was mediated through autophagy [132].

Efforts have also been made to potentiate proteasomal degradation of mutant polyQ proteins, such as through treatment with the ROCK inhibitor H1152. In SCA3 mice, intraperitoneal injection of H1152 successfully improved the neurological phenotype of the mice through reduced mutant ataxin-3 protein levels in the cerebellum, pontine nuclei, and the spinal cord [121]. Taken together, many efforts have been made to stimulate autophagy and protein clearance in an effort to diminish toxicity of the mutant proteins underlying the SCAs. Though success has certainly been achieved in cell and animal models, translation toward a suitable clinical drug is still lacking. This is in part due to issues with the compounds tested so far that are, in many cases, not suitable for direct use in patients and are also not specific for the expanded polyQ proteins. Additionally, the models used in the study to test these compounds may overestimate the contribution of autophagy due to overexpression of the mutant proteins with very long repeat sizes.

### Inhibiting Generation of Toxic Protein Fragments

For several of the polyQ disorders, a body of evidence exists supporting the notion that generation of intracellular toxic protein fragments is involved in the pathogenicity. The general understanding is that proteolytic cleavage of the mutant polyQ protein results in generation of short polyQ-containing protein fragments that result in a greater toxicity than the intact full-length protein. Evidence for this hypothesis has been found for HD [151–153], SCA3 [49, 154–158], SCA6 [159], and SCA7 [160–162].

A relation between proteolytic cleavage and generation of aggregates has been shown most convincingly for SCA3. Inhibition of the proteolytic enzyme calpain-2 in cell culture experiments was sufficient to prevent not only generation of the polyQ cleavage fragments but also the aggregation of mutant protein [163]. The cleavage of mutant ataxin-3 is likely to initiate the aggregation, after which both expanded and nonexpanded ataxin-3 are sequestered into the aggregates [164]. This observation was reproduced in SCA3 neurons derived from induced pluripotent stem cells (iPSCs), in which calpains were determined to be essential for ataxin-3 cleavage and subsequent induction of polyQ aggregates [49]. Although the aggregates are a hallmark of the diseases, they are not clearly correlated with cellular toxicity. Convincing evidence exists that at least the short polyQ-containing fragments generated through proteolytic cleavage are toxic [154]. For these reasons, preclinical research has been performed to find strategies capable of preventing generation of these toxic polyQ protein fragments. One obvious way to prevent generation of the toxic fragments is through inhibition of the proteolytic enzymes responsible for the cleavage of the polyQ-containing proteins.

In this context, the orally administered calpain inhibitor BDA-410 was tested during an 8-week period in a mouse model for SCA3. This treatment resulted in a 38% reduction in the number of mutant ataxin-3 inclusions in the striatum. Importantly, cerebellar cell loss was prevented, and an improvement in the motor phenotype of the mice could be achieved [158]. A second well-investigated way to inhibit calpains is by treatment with calpeptin, a cysteine protease inhibitor. *In vitro* experiments revealed that calpeptin treatment was able to prevent generation of ataxin-3 cleavage fragments and aggregates in iPSC-derived SCA3 neurons, proving the validity of this approach [49]. Furthermore, calpeptin proved protective in a SCA3 zebrafish model by induction of autophagy, which led to complete removal of the ataxin-3 protein [165]. Additionally, when overexpressing the endogenous calpain inhibitor calpastatin, ataxin-3 fragmentation was prevented and neuroprotective effects were established in a SCA3 mouse model [166]. This strategy was further supported by an additional study where knockout of calpastatin led to increased nuclear aggregates and aggravated neurodegeneration in SCA3 mice [156]. For HD, results from mouse models also indicated that inhibition of calpain cleavage can alleviate cellular toxicity of the polyQ protein and that this approach may thus constitute a viable therapy for polyQ disorders [167].

Despite these promising preclinical research efforts, no inhibitors preventing the formation of the toxic polyQ fragments have been assessed in clinical trials. One of the issues is that inhibiting a proteolytic enzyme will not only affect the targeted polyQ protein but will also affect the proteolytic cleavage of a wider range of proteins, and this could have unwanted side effects. Furthermore, for most of the polyQ proteins, the proteolytic enzymes that are involved in mediating toxicity for the various polyQ disorders are not conclusively identified [168]. For calpains, establishing isoform-specific inhibitors that are required to prevent side effects have been proven difficult when moving toward the clinic [169]. Similarly, caspase inhibitors have been investigated for clinical application, but many studies have been suspended due to side effects. As such, the path to caspase-targeted therapy is currently not clear [170]. Future developments may provide compounds capable of achieving isoform specificity for the various proteolytic enzymes, in turn providing better feasibility to the cleavage inhibition strategy as a potential treatment for the polyQ SCAs.

### Correcting Transcriptional Dysregulation

One well-established effect of the mutant polyQ proteins is that these proteins lead to transcriptional dysregulation. Many of the polyQ-containing proteins function as transcription factors, and evidence for a regulatory role of the polyQ repeat in transcription factors has been found [171]. This implicates that abnormal elongation of the repeat hampers this function, leading to alterations in transcription of target genes. Also, co-aggregation of other transcription factors that harbor a polyQ stretch, such as CBP [172], could contribute to this transcriptional dysregulation. Indeed, ataxin-1 knockout mice revealed overlapping alterations in cerebellar gene expression changes with Atxn1 154Q SCA1 mice, indicative of a loss-of-function effect [59]. In contrast, a similar study comparing SCA3 transgenic mice with ataxin-3 knockout mice did not find an overlap in transcriptional alterations [173]. Especially, the shorter polyQ fragments generated through proteolytic cleavage are known to translocate to the nucleus, where these fragments may interfere with transcription [174]. For instance, polyQ bound to factors such as TAFII130 interferes with CREB-dependent transcription [175]. Additionally, expanded TBP showed enhanced interaction with general transcription factor IIB, leading to downregulation of neuroprotective factor heat shock protein HSPB1 in SCA17 [176]. As such, a range of compounds aimed at reversing the transcriptional abnormalities has been tested (reviewed in [177]).

Histone acetylation and deacetylation is a process critical for the regulation of gene expression. As such, a study with transgenic SCA3 mice revealed that inhibiting histone deacetylases (HDACs) through sodium butyrate treatment was able to reverse the pathogenic histone hypoacetylation and transcriptional downregulation in the cerebellum of the mice [120]. Additionally, the motor symptoms were successfully ameliorated through sodium butyrate treatment [120]. Similar observations were made in SCA3 cell models, where the HDAC inhibitors valproic acid [178] as well as divalproex sodium [179] and sodium valproate [180] attenuated cellular toxicity induced by mutant ataxin-3. For SCA7, the HDAC inhibitor trichostatin A was tested in astrocytes expressing mutant ataxin-7 and was able to partially restore aberrant transcription of reelin, a factor involved in synaptic plasticity [181]. Based on the promising effects in cell and animal models, a randomized, double-blind, placebo-controlled trial was performed in 36 SCA3 patients to test the efficacy of the HDAC inhibitor valproic acid. Daily dosing of valproic acid was performed during 12 weeks, and at the end of the study, a significant improvement in the SARA score was observed. Some adverse effects related to dizziness and appetite were noted, but the overall conclusion was that valproic acid is a potentially useful treatment for SCA3 and that further clinical research is warranted [182]. Recent preclinical research has, however, called into question the suitability of valproic acid, as this HDAC inhibitor did not improve motor phenotype or the ataxin-3 inclusions and astrogliosis in the CMV MJD135 mouse model of SCA3 [126]. Taken together, therapies aimed at correcting the transcriptional dysregulation in the polyQ SCAs are subject to several limitations. Firstly, it is not currently established to what extent the transcriptional dysregulation is a key component of polyQ toxicity and whether targeting this pathway is enough to achieve clinically meaningful effects. Secondly, the currently tested drugs have pleiotropic effects and potential long-term side effects have not yet been adequately established.

### Other Pharmacological Targets

PolyQ disorders have a complex pathology where many cellular processes are suggested to be involved. As such, therapeutic interventions in cell and animal models targeting a range of different pathways have been suggested (reviewed in [183]). One approach has been to correct aberrant neuronal calcium signaling, a pathway suggested to be effected in multiple neurodegenerative diseases, including the polyQ disorders [184]. In SCA3, mutant ataxin-3 is able to associate with an intracellular calcium release channel. Treatment of transgenic SCA3 mice with dantrolene, a stabilizer of calcium signaling, resulted in improved motor performance and decreased neuronal cell loss [118]. However, in the treatment of the polyQ SCAs, few other studies have been performed regarding calcium signaling stabilization, and more evidence in favor of this therapeutic strategy is required before pursuing clinical trials.

Another interesting pharmacological approach for the treatment of polyQ SCAs has been resveratrol, a compound that is thought to activate SIRT1. Treatment of SCA3 mice with resveratrol improved the motor function [129]. Although the neuroprotective effect of SIRT1 activation is not fully understood, the neuroprotection could result from inhibition of neuro-inflammation and induction of autophagy [129]. Some clinical benefit of resveratrol has been observed in an open label study of Friedreich ataxia, indicating that further clinical research of resveratrol for treatment of SCA is warranted [185].

A third approach with some preclinical success is treatment with caffeine. In a SCA3 mouse model expressing mutant ataxin-3 in the striatum of 1 hemisphere, caffeine was tested by administration through the drinking water. The neuronal dysfunction and damage was successfully reduced in mice that received caffeine [122]. Later, the same research group tested caffeine in a different SCA3 mouse model, where alleviation of the behavioral deficits was achieved [186]. The mechanism is thought to involve adenosine receptor antagonism, as these receptors are involved in synaptic viability, neuroinflammation, and neuronal apoptosis [122].

Activation of serotonergic signaling has also been tested as potential treatment for SCA3. The serotonin reuptake inhibitor citalopram was recently identified in a small molecule screening as a compound capable of reducing aggregation of mutant ataxin-3 in a *Caenorhabditis elegans* model [125]. When tested in the CMV MJD135 mouse model of SCA3, citalopram strikingly reduced the motor symptoms, as well as the molecular hallmarks of SCA3, such as neuronal inclusions and astrogliosis in the mouse brain [125]. The mechanism behind this treatment may be the same as identified in HD models, where serotonin reuptake inhibition led to increased levels of brain-derived neurotrophic factor (BDNF) and enhanced neurogenesis [187].

Furthermore, the anti-glutamatergic compound riluzole was identified to improve ataxia symptoms in a randomized, double-blind, placebo-controlled pilot trial with 40 ataxia patients of different etiologies [188]. Given the proposed role of glutamatergic signaling for the induction of toxicity in SCA3 [49], riluzole was assessed in a SCA3 mouse model [128]. However, treatment with riluzole in the drinking water during 10 months did not provide an improvement in the rotarod performance, home cage activity, or body weight [128]. Surprisingly, riluzole-treated animals showed a higher number of damaged Purkinje neurons. These paradoxical results were hypothesized to be due to an excessive influx of Ca^2+^, in turn inducing increased ataxin-3 protein accumulates that are toxic to the Purkinje cells. Together, these results indicate that caution should be taken when further assessing clinical application of riluzole for the polyQ SCAs [128].

It was also shown that interferon-β treatment was capable of inducing clearance of ataxin-7 in a knock-in mouse model, resulting in improved motor function. Interferon-β induces expression of promyelocytic leukemia (PML) protein, resulting in formation of PML nuclear bodies and decreased neuronal inclusions of ataxin-7. It was therefore concluded that interferon-β is a promising molecule requiring further investigation into its suitability in treatment of SCA7 and the other polyQ disorders [131].

## Gene Therapies

**Fig. 2:**
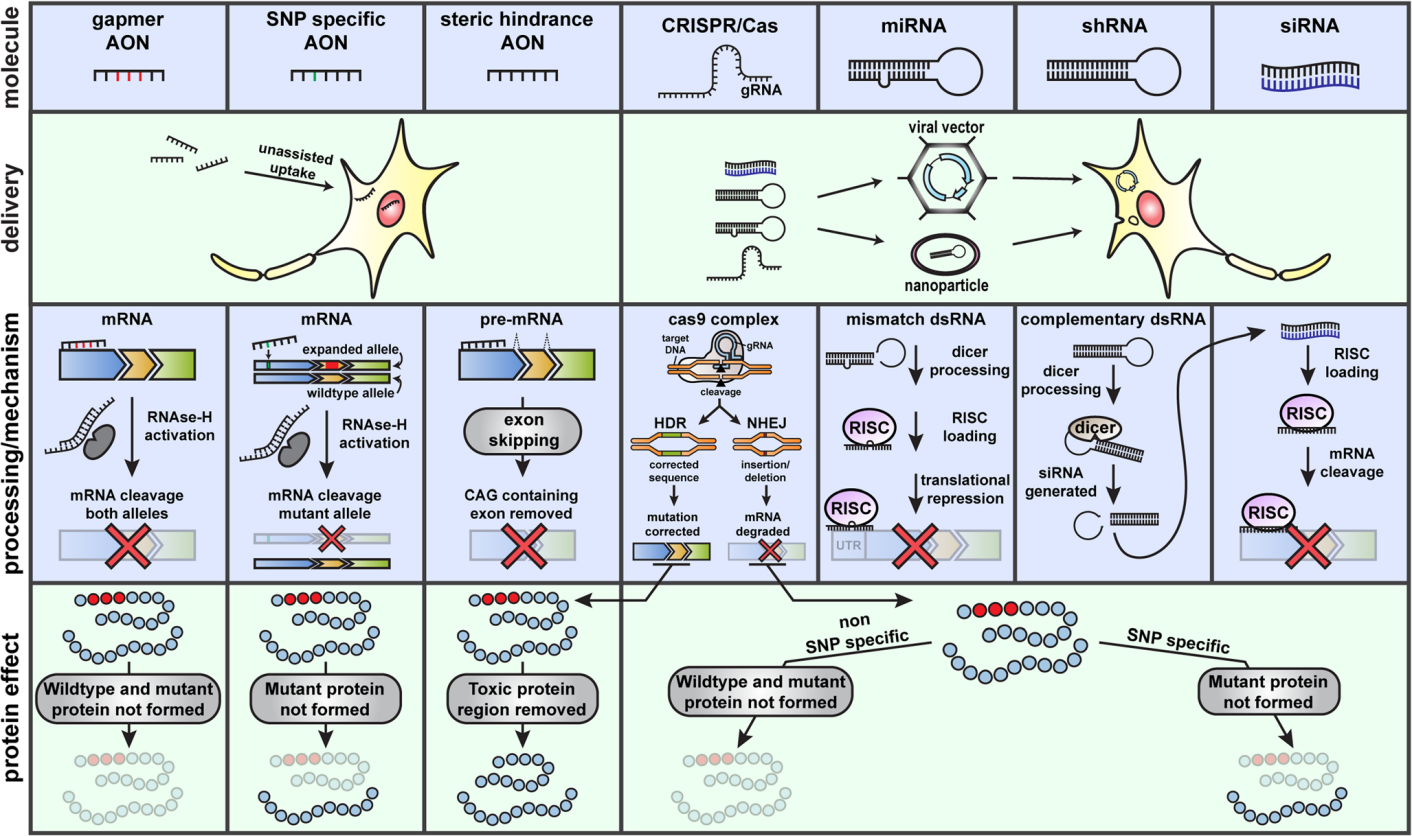
Potential genetic therapies for the polyQ SCAs. Several different nucleic acid-based molecules (top panel) are available to target the RNA or DNA of the polyQ-associated genes. The different therapeutic molecules differ in their chemical composition, delivery method, and functional mechanism. AONs can be delivered to the central nervous system as naked molecules, since their distribution, uptake, and stability in this context are excellent. CRISPR/Cas and double-stranded RNA molecules require supportive delivery methods, such as viral vectors or lipid nanoparticles. Assisted delivery of these types of molecules can be performed using different viruses, where nonintegrative gene therapy vectors based on AAV are usually preferred to avoid random integration and mutagenesis. AONs can be used to induce mRNA degradation (gapmer AONs), which activate RNase H due to formation of an RNA–DNA hybrid. Alternatively, fully 2′O-modified AONs do not activate RNase H and can be implemented to affect splicing and remove the CAG-containing exon. Downregulation of target transcripts can also be achieved through miRNA, shRNA, or siRNA. miRNA is generally designed with a mismatch, resulting in translational inhibition. shRNA and siRNA act through the same RISC pathway to degrade target mRNA. CRISPR/Cas is the most recent genetic therapy and the only strategy listed here that is able to target DNA. It can be used to inhibit expression by introducing insertions/deletions through nonhomologous end joining (NHEJ) or can introduce a corrected DNA sequence through homology-directed repair (HDR). In principle, all the mentioned molecules can be used to target SNPs associated with the pathogenic allele, resulting in downregulation or correction of the mutant allele. AON = antisense oligonucleotide, CRISPR/Cas = clustered regularly interspaced short palindromic repeats/CRISPR-associated nuclease, dsRNA = double-stranded RNA, gRNA = guide RNA, HDR = homology-directed repair, miRNA = microRNA, mRNA = messenger RNA, NHEJ = nonhomologous end joining, RISC = RNA-induced silencing complex, shRNA = short hairpin RNA, siRNA = small interfering RNA, SNP = single nucleotide polymorphism

Knockdown or modification of the polyQ genes by RNA interference (microRNA (miRNA), short hairpin RNA (shRNA), and small interfering RNA (siRNA)), antisense oligonucleotides, or DNA editing techniques (clustered regularly interspaced short palindromic repeat (CRISPR)/CRISPR-associated nuclease 9 (Cas9)) can reduce the levels of mutant expanded polyQ proteins, which will have an effect on all downstream pathological pathways (Fig. 2). Specifically targeting the disease-causing genes has the advantage of reducing the chance of nonspecific side effects but will require a unique approach for every disease. Here, we will provide an overview of the different gene therapies which are currently explored in the polyglutamine SCA field (Table 3).

**Table 3:** Gene therapies

SCA	Mechanism	Model system	Outcome	Reference
SCA1	miRNA: miR-19, miR-101, and miR-130	MCF7 and HEK293T cells	± 60% reduction of ataxin-1 protein	[189]
SCA1	miRNA: miR-144 and miR-101	HEK293T cells	20–30% reduction of ataxin-1 protein	[190]
SCA3	miRNA: *ban* miRNA	*Drosophila*	Suppression of neurodegeneration	[191]
SCA3	miRNA: miR-34	*Drosophila*	Reduced inclusion formation; the protein retained greater solubility, and neural degeneration was suppressed	[192]
SCA3	miRNA: mir-9, mir-181a, and mir-494	SCA3 mice	Reduction of ATXN3 levels, aggregate counts, and neuronal dysfunction	[193]
SCA6	miRNA: miR-3191-5p	SCA6 KI mice	Alleviation of motor deficits and Purkinje cell degeneration	[194]
SCA7	miRNA: miR-124	N2A cells and SCA7 mice	± 80% reduction of ataxin-7	[195]
SCA1	shRNA: ataxin-1 downregulation	SCA1 mice	No efficiency data on RNA or protein level; improved motor coordination, restored cerebellar morphology, and resolved characteristic ataxin-1 inclusions in Purkinje cells	[196]
SCA1	Artificial miRNA harboring a siRNA	SCA1 KI mice	58–72% reduction of both ATXN1 and Atxn1; improvement of rotarod performance and neuropathology	[197, 198]
SCA1	Artificial miRNA harboring a siRNA	B05 transgenic SCA1 mice	Improved behavior paradigms and neuropathology	[198, 199]
SCA1	Artificial miRNA harboring a siRNA	Rhesus monkeys	≥ 30% reduction of ATXN1 mRNA levels	[200]
SCA3	Artificial miRNA harboring a siRNA	HEK293 cells and MJD84.2 SCA3 mice	± 75% reduction of ataxin-3 levels (*in vitro* and *in vivo*); alleviation of nuclear accumulation of mutant ataxin-3	[201]
SCA3	Artificial miRNA harboring a siRNA	MJD84.2 SCA3 mice	Lifelong suppression of ATXN3 in the cerebellum; no mitigation of motor impairment and prolonged survival	[202]
SCA3	siRNA	SCA3 mice	Reduction of both behavior deficits and neuropathology	[203]
SCA7	Artificial miRNA harboring a siRNA	SCA7 mice	≥ 50% reduction of mutant and wild-type ataxin-7	[204]
SCA7	Artificial miRNA harboring a siRNA	SCA7 mice	Improvement of ataxia phenotypes and a reduction in cerebellar molecular layer thickness and nuclear inclusions	[205]
SCA3	siRNA: allele-specific downregulation	COS-7 and HeLa cells	> 90% reduction of mutant ataxin-3 and 25% reduction of WT ataxin-3	[206]
SCA3	siRNA: allele-specific downregulation	HEK293T cells	96% reduction of mutant ataxin-3 and 6% reduction of WT ataxin-3	[207]
SCA6	siRNA: allele-specific downregulation	HEK293T cells	> 90% reduction of the mutant protein and no reduction of WT levels	[208]
SCA7	siRNA: allele-specific downregulation	SCA7 patient-derived fibroblasts	More efficiently silencing of mutant transcript, but allele selectivity is lost at the highest dose of siRNA	[209]
SCA3	shRNA: allele-specific downregulation	SCA3 rat model	Mitigated neuropathological abnormalities	[210]
SCA3	shRNA: allele-specific downregulation	SCA3 mice	Alleviation of motor and neuropathological phenotypes	[211, 212]
SCA3	AON: ataxin-3 downregulation	MJD84.2 SCA3 mice	30% reduction mutant ataxin-3; over 75% reduction in ataxin-3 oligomers; strong improvement of motor phenotype	[213, 214]
SCA3	AON: allele-specific downregulation by targeting CAG repeat	SCA3 fibroblasts	Complete downregulation of ataxin-3 protein, with preferential targeting of mutant protein	[215, 216]
SCA3	AON and ss-siRNA: allele-specific downregulation by targeting CAG repeat	SCA3 fibroblasts	Complete downregulation of ataxin-3 protein, preferential targeting of mutant protein	[217]
SCA1 SCA3	AON: allele-specific downregulation by targeting CAG repeat	Fibroblasts	Reduction of ATXN1 and ATXN3 mutant allele at RNA; other ataxin RNAs not tested	[218]
SCA2	AON: ataxin-2 downregulation	ATXN2-Q127 and BAC-Q72 SCA2 mice	Up to 75% reduction of ataxin-2 protein in Purkinje cells of the mouse brain and significant improvement of motor phenotype	[219]
SCA7	AON: allele-specific ataxin-7 downregulation	SCA7 fibroblasts	Mutant ataxin-7 reduced up to 50% and UCHL1 expression restored	[220]
SCA3	CRISPR/Cas9	Neurons derived from patient-specific iPSCs	Successful removal of polyQ-encoding region; the ubiquitin-binding capacity of ATXN3 was retained	[221]

HEK = human embryonic kidney; MCF7 = Michigan Cancer Foundation-7; COS-7 = CV-1 in Origin Simian-7; miRNA = microRNA; siRNA = small interfering RNA; shRNA = short hairpin RNA; AON = antisense oligonucleotide

### RNA Interference

RNA interference (RNAi) is a process of sequence-specific, post-transcriptional gene silencing initiated by double-stranded RNA that is homologous to the target gene, whereby RNA molecules inhibit gene expression or translation by targeting messenger RNA (mRNA) molecules. In 1998, Fire et al. [222] discovered that RNAi could be used to manipulate gene expression in *C. elegans*, and 3 years later, Elbashir et al. [223] extended this research to include mammalian cells. RNAi can be mediated through miRNA, siRNA, or shRNA. All types of RNAi can be used to achieve similar functional outcomes, but miRNAs, siRNAs, and shRNAs are functional different molecules (Fig. 2) [224–226]. Most of the protein-coding genes in the human genome are regulated by miRNAs, which can mediate both transcriptional gene activation (TGA) and transcriptional gene silencing (TGS) [227]. In short, after transcription, primary miRNA is cleaved by Drosha to form precursor miRNA (pre-miRNA). These pre-miRNAs are transported to the cytoplasm and processed by Dicer into a mature miRNA duplex. The miRNA antisense strand is loaded into the RNA-induced silencing complex (RISC). This active complex can scan for complementary mRNAs, which subsequently can be cleaved and degraded [228]. In case there is a single nucleotide mismatch in complementarity between the miRNA and its target, this will lead to translational repression [229] (Fig. 2). siRNAs are chemically synthesized double-stranded RNAs. Upon entering a cell, siRNAs are recognized by the RISC. Next, the siRNA is unwound to form single-stranded siRNA which is part of RISC, where it follows the same pathway as described before (Fig. 2). shRNAs are vector-based and can be delivered into mammalian cells through viral vectors or nanoparticles, allowing stable integration and long-term knockdown of the targeted gene. shRNAs consist of RNA sequences linked by a short loop. After transcription, the shRNA sequence is exported to the cytosol and recognized by Dicer. Dicer subsequently processes the shRNA into siRNA duplexes, and it follows a similar pathway as the chemically synthesized siRNAs [230] (Fig. 2).

### miRNA-Based Therapies

In the polyQ SCA field, several miRNAs have been identified that regulate the polyQ genes causing SCA that could provide potential therapeutic targets [231]. By using miRNA target prediction databases, several evolutionarily conserved miRNA-binding sites in the 3′ UTR of the human *ATXN1* gene were found [232]. These miRNAs were validated in MCF7 cells. Furthermore, inhibition of the miRNA-mediated post-transcriptional regulation of *ATXN1* caused severe cytotoxicity in a SCA1 cell model [189]. Another SCA1 study found age-related differences in miRNA expression levels in the cortex and cerebellum of humans and nonhuman primates on a genome-wide scale. Moreover, this study found that miR-144 and miR-101 inhibition increased ATXN1 levels in a human cell model, concluding that activation of miRNA expression may protect from cytotoxicity caused by ATXN1 [190]. In *Drosophila* models, miRNA expression studies were performed for SCA1, SCA2, SCA3, and SCA7. Various miRNAs were found to be (almost) differentially expressed and could be potential targets for a miRNA-based therapy [191, 192, 233, 234]. In a SCA3 animal model and human neurons, 3 miRNAs were identified that interact with the ATXN3 3′ UTR and whose expression is dysregulated. Injecting lentiviral vectors encoding for these miRNAs in the striatum of 5-week-old SCA3 mice resulted in reduction of ataxin-3 levels and nuclear aggregates and improvement of neuronal dysfunction [193]. Similar results have been obtained with adeno-associated virus (AAV)-mediated delivery of miR-3191-5p in SCA6 mice. Viral delivery of this miRNA ameliorated behavioral deficits and Purkinje cell degeneration [194]. Finally, in a mouse neuroblastoma cell line and a SCA7 mouse model, it has been found that miR-124 mediates the interaction of lnc-SCA7 and Atxn7 transcripts. Delivery of miR124 mimics decreased the expression of *Atxn7* [195]. Altogether, these findings suggest that dysregulation of miRNA pathways can be found in the polyQ SCAs and that restoring expression of these miRNAs can improve disease phenotypes. However, the fact that 1 miRNA can target many other transcripts [235] and cause unwanted off-target effects is a major drawback to take miRNA-based therapies to the clinic.

### shRNA- and siRNA-Based Therapies

The first use of shRNA as gene-specific therapeutics in the polyQ SCA field was published in 2004. Upon intracerebellar injection of recombinant AAV vectors expressing shRNAs, cerebellar Purkinje cells were successfully transduced. No data on the percentage of reduction was shown, but treated animals showed improved motor function, restored cerebellar morphology, and resolved ataxin-1 inclusions in Purkinje cells in SCA1 mice [196]. Keiser et al. [197–199] took advantage of improvements in both expression systems and siRNA design and performed experiments in different SCA1 mouse models. Upon bilateral injection into the DCN, SCA1 mice showed improvement in both behavioral and neuropathological phenotypes [197–199]. Furthermore, after a single injection, adult rhesus macaque showed reduction of endogenous ATXN1 mRNA levels in the DCN, cerebellar cortex, inferior olive, and thalamus compared to the uninjected hemispheres. No clinical complications were observed, and quantitative and qualitative analyses suggest that this therapeutic intervention strategy and subsequent reduction of ATXN1 are well tolerated [200]. Altogether, these preclinical data are supportive of a clinical application of AAV-based siRNA therapy based on artificial miRNAs in SCA1 [196, 197, 199, 200]. For SCA3, AAV-mediated delivery of siRNAs targeting the 3′ UTR of *ATXN3* to the cerebellum of a humanized SCA3 mouse model leads to gene silencing of human mutant ATXN3 [201, 202]. Short-term treatment cleared the nuclear accumulation of mutant ataxin-3 throughout the cerebellum [201], but long-term treatment did not reverse motor impairment or survival phenotypes [202]. These results suggest that targeting a large extent of the cerebellum may not be sufficient for an effective clinical trial in SCA3 patients [202]. Interestingly, Conceição et al. [203] used systemic administration of nonviral, stable nucleic acid lipid particles (SNALPs), incorporating a short peptide derived from rabies virus glycoprotein and encapsulating the siRNAs targeting the mutant ataxin-3. The authors reported efficient silencing of the mutant ataxin-3 and reduction of both behavior deficits and neuropathology in SCA3 mouse models. This study was the first to report that a noninvasive, systemic administration was beneficial in a preclinical study of a polyQ disorder [203]. A ≥ 50% reduction of both mutant and wild-type ataxin-7 is reported after viral siRNA delivery in a SCA7 mouse model. Although this mouse model expresses the mutant transgene in the retina, no obvious retinal phenotype is seen. Nevertheless, upon treatment, the normal retinal function is preserved and no toxicity has been reported [204]. Furthermore, this research group demonstrated a significant improvement of ataxia phenotypes and a reduction in cerebellar molecular layer thickness and nuclear inclusions [205], concluding that the reduction of ataxin-7 is well tolerated without any adverse toxicity [204, 205].

Above-described strategies are promising but have the disadvantage that both wild-type and mutant genes are simultaneously downregulated. A promising RNAi strategy to overcome this problem is to suppress the expression of only the expanded polyQ genes implicated in the polyQ SCAs. A selective reduction in the expression of the expanded genes can be achieved by exploiting differences in nucleotide sequence between the expanded polyQ alleles and the wild-type polyQ alleles. This strategy holds great promise for the treatment of polyQ SCAs, since the function of the wild-type allele is maintained [236]. In SCA3, a single nucleotide polymorphism (SNP) linked to the expanded polyQ allele [237] is used in this manner. Research demonstrated that in SCA3 cell models, allele-specific silencing of *ATXN3* can be achieved by targeting this linked SNP by siRNAs [206, 207]. Studies in cell models for SCA6 and SCA7 showed that a similar siRNA approach was successful in the knockdown of these mutant transcripts. Moreover, there was only a modest reduction of the WT ataxin levels [208, 209]. Furthermore, allele-specific RNAi using shRNA showed improvement of motor and neuropathological deficits in SCA3 mouse and rat models [210–212]. Recently, the FDA approved the first-ever RNAi therapeutic, patisiran, for the treatment of polyneuropathy of hereditary transthyretin-mediated amyloidosis [238]. These results paved the way for the first clinical trial in polyQ SCA patients.

### Antisense Oligonucleotides

One category of gene therapy that has made great progress over the last decades is antisense oligonucleotides (AONs). AONs are short, synthetic, single-stranded strings of nucleic acids of approximately 8 to 50 nucleotides in length [239]. AONs are designed to interfere with RNA through a variety of mechanisms, such as interfering with splicing, or downregulation through RNase H-mediated degradation of the target transcript (Fig. 2) [240]. For monogenetic disorders such as the polyQ SCAs, AONs are a promising therapeutic tool for a variety of reasons. Firstly, the genetic target for the polyQ SCAs is known, as the responsible gene and location of the toxic CAG repeat have been well described [40]. Secondly, recent years have shown great promise for AON therapies in application for neurodegenerative disorders. The first preclinical experiments using AONs to treat a neurodegenerative disorder were performed in 2006. AON treatment significantly slowed disease progression in a rat model of amyotrophic lateral sclerosis (ALS) caused by a SOD1 mutation [241], followed, 7 years later, by the first clinical trial of intrathecal delivery of an AON in SOD1 patients. No serious adverse events occurred in patients, and re-enrolment and retreatment were also well tolerated [242]. The successful delivery in the cerebrospinal fluid (CSF) and the outstanding safety profile paved the way for AONs as treatment for other neurodegenerative diseases. In 2009, experiments in an animal model for spinal muscular atrophy (SMA), a rare neuromuscular disorder characterized by a loss of motor neurons and progressive muscle wasting, show that AONs that abrogate aberrant splicing of SMN2 are promising compounds for treating SMA [243]. Most notably, this AON-based drug named Spinraza recently received FDA and EMA approval for treatment of SMA [244], following favorable outcomes in a phase 3 clinical trial where the treated children showed improved survival compared to placebo [245]. Upon intrathecal injection, AONs distribute well throughout the CNS and are taken up efficiently by cells of the brain. Also, the AONs remain present and stable in the tissue for months, allowing for infrequent dosing [246, 247]. In 2012, it has been demonstrated for the first time in a polyglutamine disease that infusion into the CSF of HD mouse models delays disease progression [248]. Preliminary results from a phase 1b/2a clinical trial following this preclinical research have already been released by Ionis Pharmaceuticals, with reports stating that the safety and tolerability profile are favorable and warrant further development [249]. Additionally, dose-dependent reduction in huntingtin protein levels in the CSF was reported [250]. Given that the similar approach and mechanism, it can be assumed that similar AONs for application for the polyQ SCAs can be equally well tolerated.

Given their favorable therapeutic properties, AONs clearly hold promise for the treatment of the polyQ SCAs. As such, several AON-based therapies are currently under research to this end, in particular for SCA3. The most straightforward approach is to target the mutant transcript for downregulation. This can be achieved either allele specifically for the transcript containing the expanded CAG repeat or nonallele specifically by targeting both mutant and wild-type transcripts.

#### Allele-Specific Downregulation

In order to specifically target the mutant allele, the CAG repeat can be directly targeted with AONs that preferentially bind to the longer repeat. Such AONs have been tested for SCA3, where peptide nucleic acids (PNAs) and locked nucleic acid (LNA) AONs were designed complementary to the CAG repeat and tested in different cell lines. Several of the tested AONs were indeed capable of downregulating mutant ataxin-3 at both transcript and protein levels [215]. The authors then tested a new range of AONs and showed several candidates capable of preferentially targeting the mutant ataxin-3 protein [216]. More recently, the same group has investigated a large range of new CAG repeat-targeting AONs with combinations of different chemical modifications and mismatches for downregulation of mutant ataxin-3. The authors found that subtle changes to the composition of the AON led to significant effects on efficacy and allele specificity. Through the screening of many candidates, the researchers identified several very promising AONs capable of potently downregulating mutant ataxin-3 in cells through CAG targeting [217]. Similar repeat-targeting AONs were tested in SCA7 cell lines, where ataxin-7 downregulation was achieved with varying efficiency based on the specific chemistry used [220]. To our knowledge, the CAG repeat-targeting AONs have not yet been tested in animal models of SCA, but positive results have been obtained where these types of AONs were injected in the brains of HD mice [251]. Specifically, CAG repeat-targeting AONs reduced mutant huntingtin (HTT) protein with 15 to 60% throughout the mouse brain, and corresponding improvements in motor tasks were reported [251]. These studies thus suggest good promise for these particular CAG repeat-targeting AONs for application in the polyQ SCAs, and indeed the other polyQ disorders [218].

Apart from targeting the CAG repeat, the mutant allele can contain SNPs that can be useful for specific targeting with AONs. The SNP targeting strategy is also largely investigated in the context of HD, where especially the newer AON chemistries show good potential of targeting the mutant allele [252, 253]. For instance for SCA3, a SNP that is associated with the mutant allele in 70% of SCA3 patients has been described [237] and has been proven as a viable target for targeting with shRNA [210]. A similar strategy has been described for SCA7 [209]. Despite these findings, no AON-mediated approach targeting the SNPs has been described for the polyQ SCAs to date.

#### Nonallele-Specific Downregulation

Specific downregulation of only the mutant allele may not be viable for all polyQ SCAs, and therefore, nonallele-specific downregulation, in which both alleles are targeted, may be a more achievable approach. An important consideration when pursuing this approach is whether prolonged efficient downregulation of the targeted protein will not have a detrimental effect, since most of the proteins that cause polyQ SCAs have important cellular function. A screening of AONs for downregulation of *ATXN3* yielded several candidates capable of reducing expression with 50% [213]. Subsequent testing in SCA3 mice showed that the AON distributed well throughout the mouse brain and was capable of alleviating the motor symptoms in addition to preventing nuclear accumulation of mutant ataxin-3 for at least 14 weeks in the mouse brain [214]. Taking into account the level of ataxin-3 overexpression in the homozygous SCA3 mouse that was used, the lead AON is an exciting candidate for further clinical advancement [213]. Similarly, AONs have been designed to target ataxin-2 for downregulation. In a recent seminal study, again by screening a large number of different AON sequences, researchers were able to find an AON capable of potently downregulating ataxin-2 in cells. Intracerebroventricular injection of this AON in 2 different SCA2 mouse models yielded potent suppression of ATXN2 in the cerebellum. As a result, the motor phenotype of the SCA2 mice was markedly improved after AON injection [219]. Though the therapeutic AON candidates for the polyQ SCAs are currently still in preclinical phase, some speculation on the applicability of AONs for this purpose can already be made. For instance, an RNase H-based AON for downregulation of huntingtin is currently undergoing clinical trial. The AON is capable of downregulating both huntingtin alleles and has shown strong efficacy in preclinical research [248].

#### Exon Skipping

The polyQ SCAs are all caused by the same type of mutation, the expansion of a CAG repeat in the coding region of the responsible gene. For this reason, an interesting AON-based approach is to exclude the exon containing the CAG repeat from the transcript. This can be achieved through an approach termed exon skipping, where specific splicing signals are masked by AONs [254]. If removal of the exon in question does not result in a reading frame shift, the mRNA transcript is not degraded and can be translated in a normal fashion. This manner of splice modulation with AONs is a particularly useful strategy for application in the brain [239, 255]. Nonetheless, AON-mediated removal of the CAG repeat is not yet widely investigated for the polyQ disorders. This is in part due to the fact that the target gene needs to meet certain requirements. For instance, in *ATXN2*, the CAG repeat is located in the first exon and skipping of this exon would thus remove the start codon, resulting in downregulation of the transcript [23].

In this regard, *ATXN3* has been most extensively investigated with regard to exon skipping. Firstly, Evers et al. [256] proved that AON mediated skipping of exon 9 and the CAG-containing exon 10 from the *ATXN3* transcript led to formation of an internally truncated ataxin-3 protein lacking the polyQ repeat in SCA3 patient-derived fibroblasts. Moreover, the modified ataxin-3 protein was shown to retain its ubiquitin binding function [256]. More recent research from the same group demonstrates the possibility to skip only exon 10 from *ATXN3* pre-mRNA, which then results in formation of a truncated ataxin-3 protein lacking the polyQ repeat and C-terminal region of the protein. This strategy was subsequently tested in a SCA3 mouse model, where AON treatment led to formation of the truncated ataxin-3 protein in all tested brain regions [257]. Interestingly, researchers serendipitously discovered that targeting the CAG repeat directly with 2′O-modified AONs also led to exclusion of *ATXN3* exon 10, resulting in similar formation of a truncated ataxin-3 protein [217]. Targeting the mutant *ATXN3* allele could be preferentially targeted for exon skipping by the CAG-targeting AONs, and this might be a very favorable therapeutic approach, since only the mutation could be specifically targeted for exclusion from the transcript.

Apart from skipping the CAG repeat-containing exon, AONs can also be implemented to skip other exons that have the potential to contribute to toxicity. For instance, proteolytic cleavage of the polyQ proteins can liberate shorter, more toxic, protein fragments for HD [258, 259] and SCA3 [154]. Hence, removing the protein region containing the proteolytic cleavage sites associated with the toxic fragments would, in theory, prevent toxicity of the mutant polyQ protein. For SCA3, the approach was concluded to be suboptimal due to inefficient exon skipping and is currently no longer pursued for clinical application [260]. The splice-modulating AONs, implemented for either exon skipping or inclusion, have been well investigated over the last years, especially for application in Duchene muscular dystrophy and SMA, where Spinraza is currently the first approved AON drug for clinical application in the brain.

### CRISPR/Cas9

Advances in genome engineering techniques created the possibility to facilitate efficient genome engineering in eukaryotic cells [261–263]. The CRISPRs and Cas9 have been developed to target and edit specific genomic regions in (mammalian) cells [261, 263]. In short, guide RNAs (gRNAs) complementary to specific genomic sites are used to target Cas9 and induce double-stranded breaks (DSBs), which can be repaired through error-prone nonhomologous end joining (NHEJ) or the high-fidelity homology-directed repair (HDR) pathway. NHEJ leads to insertions or deletions (indels), leading to a frameshift and a premature stop codon. Therefore, this process can be used to make gene-specific knockouts. HDR generates defined gene modifications due to the introduction of a repair template [263].

Recently, Ouyang et al. [221] performed CRISPR/Cas9-mediated deletion of exon 10 containing the polyQ-encoding region in SCA3 patient-derived iPSCs. The corrected lines retained pluripotency and neural differentiation capacities. Moreover, the ubiquitin-binding capacity of ATXN3 was retained [221]. In a publication investigating HD, several allele-specific gRNAs for polyQ SCAs were proposed. In this study, a personalized, allele-specific CRISPR/Cas9 strategy was successfully tested for HD [264]. First, a genomic DNA locus was screened for SNPs that generate or eliminate protospacer adjacent motif (PAM) sequences. PAM sequences in the promoter region and transcription start site that are present on the mutant allele, but absent from the normal allele, were selected, and gRNAs were designed. Next, mutant alleles were inactivated in an allele-specific manner, resulting in the absence of mutant *HTT* RNA and HTT protein containing the expanded polyQ repeat. Based on the 1000 Genomes database, the authors proposed dozens of personalized, allele-specific gRNAs that can be tested in polyQ SCA preclinical studies [264].

CRISPR/Cas9 has the potential to become a great success in clinical trials for many diseases including the polyQ SCAs. However, off-target effects, which may cause genomic instability and disrupt functionality of nontargeted genes, are still a major concern [265]. Thus far, it was thought that CRISPR/Cas9 was reasonably specific. Recently, it has been shown that repair of double-strand breaks induced by CRISPR/Cas9 leads to large deletions and complex genomic rearrangements at the target sites [266]. This is an important issue that needs to be solved before clinical trials are started. Another point of concern is the delivery to the target cells. Many delivery strategies of the CRISPR/Cas9 system have been developed and are reviewed in Liu et al. [267]. So far, it is challenging to target the nondividing neurons in the human brain. Recently, Nishiyama et al. [268] showed that genome editing via HDR was successfully induced in mitotic and postmitotic cells *in vitro* and *in vivo*. The efficiency of HDR was with approximately 10% relatively low, but similar among different brain regions. This suggests that gene editing is applicable to practically any brain region and cell type at any given time point in life [268].

## Stem Cell Therapies

Since the first stem cell-based clinical trials using fetal midbrain tissue to replace the dopamine neurons that are lost in PD [269, 270], stem cell replacement therapy has evolved and many more clinical trials were initiated [271]. Here, we give an overview of the current state of the stem cell-based therapies in the polyQ SCA field (Table 4).

**Table 4:** Stem cell-based therapies

SCA	Cell type	Delivery method	Mouse model	Results	Reference
SCA1	ESC	Stereotaxic injection into the deep cerebellar nuclei	B05 transgenic SCA1 mice	Better performance on multiple behavioral tests of cerebellar function	[272]
SCA1	Adult NPC	Stereotaxic injection into the cerebellar white matter	B05 transgenic SCA1 mice	Only in mice with significant cell loss, grafted NPCs migrated into the cerebellar cortex. Improved motor skills, a significantly thicker molecular layer, more surviving PCs, and normalization of the PC basal membrane potential	[273]
SCA3	Cerebellar NSC	Transplantation into the cerebellum	SCA3/MJD transgenic mice	A significant and robust alleviation of the motor behavior impairments, which correlated with preservation from SCA3/MJD-associated neuropathology, namely reduction of Purkinje cell loss and reduction of cellular layer shrinkage and aggregates	[274]
SCA1	MSC	Stereotaxic intrathecal injection	B05 transgenic SCA1 mice	Suppression atrophy of PC dendrites and better performance on rotarod	[275]
SCA1	MSC	Stereotaxic intrathecal injection	SCA1-KI mice	Suppressing peripheral nervous system degeneration	[276]
SCA2	MSC	Intravenous and intracranial transplantation	SCA2 transgenic mice	Intracranial transplantation: no effect Intravenous: improved rotarod performance and delayed onset of motor function deterioration	[277]
SCA3	MSC	Single intracranial injection and repeated systemic administration	SCA3 transgenic mice	Transplantation: only transient effects Periodic administration: Sustained motor behavior and neuropathology alleviation	[278]

SCA = spinocerebellar ataxia; ESC = embryonic stem cell; NPC = neural precursor cell; NSC = neural stem cell; MSC = mesenchymal stem cell

In the polyQ SCA field, the first preclinical experiment of embryonic cerebellar transplants into a transgenic mouse model of SCA1 has been published by Kaemmerer and Low [272] at the end of the previous millennium. Compared with sham-operated littermates, grafted mice functioned better on multiple cerebellar behavior tests. Improvements continued for approximately 3 months after the transplantation, after which a progressive decline in motor performance was observed. In most graft recipients, donor Purkinje cell survival was evident 20 weeks after surgery [272]. These first experiments show that transplants can survive and have behavioral benefits despite the ongoing pathological process in the diseased brain. Chintawar et al. [273] transplanted neural precursor cells (NPCs) derived from adult mice into the cerebellar white matter of SCA1 mice at different stages of the disease process. Only in mice with significant cell loss, grafted NPCs migrated into the cerebellar cortex. These animals showed behavioral benefits, a thicker molecular layer, and more surviving Purkinje cells compared to sham-treated controls. Despite these results, the grafted cells did not adopt the morphological characteristics of Purkinje cells and levels of neurotrophic factors were not increased, suggesting that the neuroprotective effect of grafted NPCs was mediated by direct contact with the host Purkinje cells [273]. Mendonça et al. [274] transplanted cerebellar neural stem cells into the cerebellum of adult SCA3 mice and found alleviation of the motor behavior impairments, which correlated with the prevention of SCA3 disease-associated neuropathology. Furthermore, a significant reduction of neuroinflammation and an increase of neurotrophic factors levels were found, suggesting that transplantation triggers important neuroprotective effects [274]. The increase in neurotrophic factors can be explained by the fact that a pool of multipotent, SOX2-positive cells remained in the graft [274].

Another cell type that is widely used in cell-based clinical trials is mesenchymal stromal cells (MSCs) [279–283]. MSCs are multipotent progenitor cells that can be isolated from multiple sources such as bone marrow [284], umbilical cord blood [285], or adipose tissue [286, 287]. Furthermore, MSCs have the ability to migrate and integrate into the endogenous neural network and subsequently produce various trophic factors involved in functional recovery, neuronal cell survival, and stimulation of endogenous regeneration [288]. In 2008, a protocol has been developed for inducing differentiation of MSCs toward neurotrophic factor (NTF)-secreting cells [289]. These cells can act as a protective agent in neurodegenerative diseases [290]. Chang et al. [277] showed that intravenous injection of MSCs in SCA2 mice improved rotarod performance and Purkinje cell survival, while intracranial transplantation failed to achieve these neuroprotective effects [277]. Intrathecal injection of MSCs and intravenous injection of MSC-conditioned medium ameliorated both cerebellar pathology and behavior phenotypes in different mouse models of SCA1 and SCA3 [275, 276, 278, 291]. The first phase 1/2 clinical trials to evaluate the safety, tolerability, and efficacy of intrathecal injection or intravenous administration of MSCs from healthy donors in polyQ SCA patients have been performed [292–295]. All these studies report that administration of MSCs is safe without any adverse events or rejection reactions. Administration of MSCs might delay the progression of neurologic deficits for polyQ SCAs, but a next, important step will be randomized, double-blind, placebo-controlled phase 2 trials.

The ground-breaking finding that somatic cells can be reprogrammed into iPSCs that can self-renew [296, 297] has enabled the development of an unlimited source of any type of human cells which can be used for disease modeling, drug discovery, and stem cell-based therapies (reviewed in [298]). All studies report a positive effect on both neuropathology and different behavior paradigms. So far, no iPSC replacement studies have been reported for the polyQ SCAs, but for HD, cell replacement therapy using iPSCs has been studied, mainly in rat models [299–301]. To date, patient-derived polyQ iPSCs are used for disease modeling and drug screens [49, 302–306]. Although the first preclinical experiments in HD and other neurodegenerative disease models are positive, an iPSC-based therapy for the polyQ SCAs is far from a clinical application.

## Future Perspectives

A wide diversity of therapies for polyQ SCAs have been tested in preclinical models, and some of these therapies have even moved to the clinical trial stage. Most of these studies show promising results in both cell-based and animal models. In polyQ SCA mouse models, improvement of disease-associated neuropathology, most importantly the reduction of Purkinje cell loss and toxic aggregates, and alleviation of the behavior deficits have been achieved. So far however, these successes have not been translated to noteworthy breakthroughs in clinical trials. This may be in part due to the relatively small patient population and slow disease progression with a large variation in symptoms that is inherent to the SCAs. Nonetheless, there are plentiful compounds and treatment strategies being investigated that show promise for treatment of the SCAs.

Arguably, the most promising and elegant strategy to cure the polyQ SCAs is replacing the expanded CAG repeat by a control repeat length using CRISPR/Cas9. In this manner, the gain-of-function toxicity caused by the expanded allele is cleared and the repaired allele results in normal expression levels of the various ataxin proteins. To date, most CRISPR/Cas9 strategies are not immediately applicable for treating polyQ SCA patients. Various technical challenges, including minimizing off-target effects and optimizing delivery in all of the targets cells, need to be solved first. Therefore, the development of genome-editing therapies that induce HDR in the mutant allele only is crucial. Given the current challenges for clinical application of CRISPR/Cas9, RNA targeting therapies will be the solution for the near future. Exciting recent results from clinical trials in various neurodegenerative diseases have shown that RNA targeting compounds such as AONs can safely and effectively enter the cells of the CNS without inducing strong side effects.

The polyQ SCAs are a group of devastating progressive disease where historically, only symptomatic treatment has been available. With research progress in recent years, however, there is great promise that a therapy to delay disease progression or even to halt disease onset can be achieved. However, caution is still needed because progressing therapeutic approaches into approved drugs has been proven to be challenging.
